# Effect of a supplement rich in alkaline minerals on acid-base balance in humans

**DOI:** 10.1186/1475-2891-8-23

**Published:** 2009-06-10

**Authors:** Daniel König, Klaus Muser, Hans-Hermann Dickhuth, Aloys Berg, Peter Deibert

**Affiliations:** 1 University Hospital Freiburg, Centre for Internal Medicine, Department of Rehabilitation, Prevention and Sports Medicine, Germany

## Abstract

**Background:**

Western diets are considered acidogenic due to the high dietary acid load and a low intake of base-forming dietary minerals such as potassium, magnesium or calcium. In the present study we investigated the effect of a multimineral supplement (MMS) rich in alkaline minerals on acute and chronic regulation of acid-base balance with the pH of blood, urine and saliva as potential surrogate markers.

**Methods:**

Parameters were measured (i) without MMS intake, (ii) in the three consecutive hours following ingestion (blood and urinary pH) and (iii) during one week with or without MMS intake (self-monitored using pH measurement strips).

**Results:**

25 (15 female; 10 male) subjects (age 44 ± 14 y; BMI 23.9 ± 1.9 kg/m^2^) were enrolled in the investigation. Following acute administration of the MMS in the morning, blood ph (1 and 2 h after ingestion) rose from 7.40 to 7.41; p < 0.05, and also urinary pH 3 h after ingestion (5.94 to 6.57; p < 0.05) increased significantly.

Following longer-term supplementation, both the increase in urinary pH in the morning and in the evening occurred within 1 day. Compared to pH values without the MMS, average pH in urine was 11% higher in the morning and 5% higher in the evening. Analyses of food records showed that the increase in urinary pH was not related to dietary change.

**Conclusion:**

Our results suggest that the ingestion of a multimineral supplement is associated with both a significant increase in blood and urinary pH. The health related consequences of this supplementation remain to be determined.

## Background

Dietary behaviour has shown to influence acid-base balance [1]. In general, western diets are considered acidogenic due to the high amount of animal protein [2,3] and an insufficient intake of fruits and vegetables. This is associated with a high dietary acid load and a low intake of base-forming dietary minerals such as potassium, magnesium or calcium [4,5].

A mismatch between acid- and base-forming nutrients may result in subclinical low-grade acidosis [6]. Several mechanisms are employed to compensate the excess in dietary acid load. One of them is the release of alkaline calcium salts from the skeleton to maintain the acid-base balance. Hypercalciuria may have several pathological consequences; amongst others, it has been hypothesized that it contributes to the pathogenesis of osteoporosis [7]. Fruit and vegetable intake provides alkaline minerals in particular potassium salts [8]. Previous investigations have reported beneficial effects of dietary potassium and potassium-rich foods or mineral water on bone health [9,10]. Although large controlled clinical trials are still scarce, there is evidence that an increased intake of base-forming nutrients may be associated with improved health outcomes [11].

In some animal models, it has been shown that supplementation with alkaline minerals neutralizes diet-induced metabolic acidosis [12] and is associated with higher bone mass. In addition, potassium bicarbonate has been shown to reduce calcium excretion in postmenopausal women in a dose dependent manner [13]. Furthermore, in 18 postmenopausal women, bone resorption was reduced and bone formation was increased following potassium bicarbonate intake [9]. Comparable results were observed in 161 postmenopausal women following supplementation with potassium citrate [14]. Apart from the release of skeletal calcium to maintain acid-base balance, it has also been shown that a low pH stimulates osteoclasts and inhibits bone matrix mineralization [15]. Therefore, several lines of evidence suggest that a correction of dietary acid load could improve acid-base balance and thereby reduce chronic disease such as osteoporosis, kidney stones or sarcopenia [5,16,17]. The (EPIC)-Norfolk population has shown that urine pH may serve as an indicator of dietary acid-base load [18]. To our knowledge, there is very limited data showing that supplementation with alkaline minerals directly influences acid-base homeostasis in humans. Therefore we investigated the effect of a mineral supplement rich in alkaline minerals on acute and chronic regulation of acid-base balance through surrogate markers of blood, urine, and saliva pH [18].

## Methods

All subjects completed a comprehensive medical examination and routine blood tests. Subjects were excluded if they showed inflammatory diseases or corresponding laboratory findings (elevated leukocytes, C-reactive protein or ESR), renal and pulmonary dysfunction or metabolic disorders, particularly disturbances in acid-base balance. Subjects with unstable dietary habits or taking supplements effecting acid-base metabolism were also excluded from the study. Participants were told to maintain their lifestyle behaviours and particularly nutritional habits throughout the study period.

The study protocol was approved by the ethical committee of the University of Freiburg and all subjects provided written informed consent.

**Figure 1:**
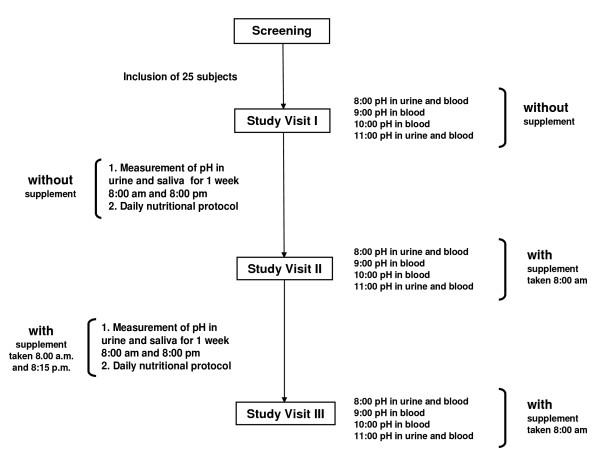
**Study design**.

The study protocol is shown in Fig. 1. The mineral supplements were provided by a German manufacturer (Basis Balance, Anton Huebner GmbH & Co. KG, Ehrenkrichen, Germany [Table 1]). Following inclusion in the study, the course of pH, pCO_2_, bicarbonate and base excess was measured at baseline without any intervention in the fasting state at 8:00, 9:00, 10:00 and 11:00 am. Urinary pH was determined at 8:00 and 11:00 am. The following day, subjects completed standardized nutritional protocols on a daily basis, which were later analyzed using a computer based software programs (DGE-PC, Version 3.1). Study participants determined their urinary and salivary pH values at 8:00 ± 1 h am before breakfast and at 8:00 pm ± 1 h before dinner with pH test paper strips. Before beginning of the study, participants had been trained to interpret the colour of the pH paper.

**Table 1:** Composition of the mineral supplement (daily dose).

**Minerals**		**Trace elements**	
Potassium	600 mg	Copper	1000 μg
Calcium	500 mg	Zinc	5 mg
Magnesium	200 mg	Iron	5 mg
Sodium	200 mg	Chromium	60 μg
		Molybdane	80 μg
		Selen	30 μg

The daily dose contains 65 mEq of alkalines (Basis Balance, Anton Huebner GmbH & Co. KG, Ehrenkirchen, Germany)

On the occasion of the next examination, study participants ingested the mineral supplement dissolved in water in the dosage provided by the manufacturer (30 g) at 8:00 am without any other food intake. Again, pH, pCO_2_, bicarbonate and base excess were measured hourly from 8:00 until 11:00 am and urinary pH was determined at 8:00 am and 11:00 am. In the following week subjects continued to fill out the food records and ingested 30 g of the mineral supplement in the morning as well as in the evening. Urinary and salivary pH values were self-measured at 8:00 ± 1 h am in the morning before breakfast and at 8:00 pm ± 1 h in the evening before dinner by pH test paper strips.

After one week, the same examination took place as described before at 8:00, 9:00, 10:00 and 11:00 am (urine and blood).

### Statistical methods

Normality of all variables was tested using the Kolmogorov-Smirnov test procedure. Testing for changes between examination at baseline and at each examination was done by paired sample T-test. All p values were two-sided and a p value of 0.05 or less was considered to indicate statistical significance. Analysis was conducted with the use of SPSS software (version 13.0).

## Results

25 (15 female; 10 male) subjects (age 44 ± 14 y; BMI 23.9 ± 1.9 kg/m^2^) were enrolled in this investigation. All subjects completed the study without relevant side effects that could be ascribed to the supplement. The supplement was well tolerated and compliance was good.

None of the subjects showed abnormal values in any parameters investigated. There were no signs of severe acute or chronic disturbances in acid base balance, neither before nor during or after the supplement intake.

**Figure 2:**
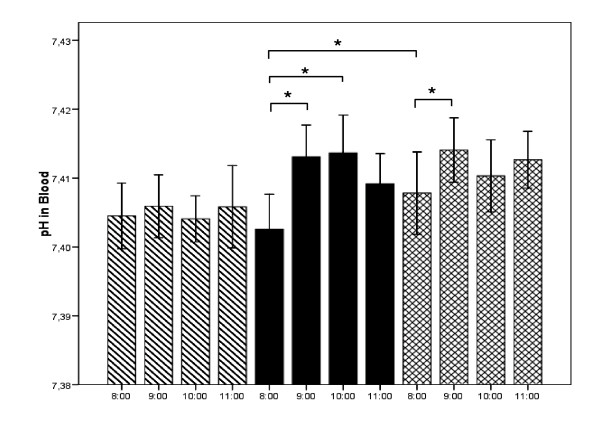
**Alterations in blood ph without intake of the multi-mineral supplement (MMS) (hatched bars), following MMS in the morning without (black bars) or with prior 1 week of chronic MMS supplementation (crossed bars)**. Values are mean values ± SEM. * = p < .05; ** = p < .01 compared to baseline value.

Alterations in blood pH are shown in Fig. 2. No significant changes were observed in blood pH measured hourly from 8 am to 11:00 am without intake of the multi-mineral supplement (MMS) (hatched bars). Following MMS in the morning, blood pH significantly increased both 1 and 2 hours after ingestion (black bars). The crossed bars demonstrate that the acute increase in pH was also detectable following 1 week of chronic MMS supplementation. In addition, baseline pH in the morning was significantly higher following one week of supplementation.

**Figure 3:**
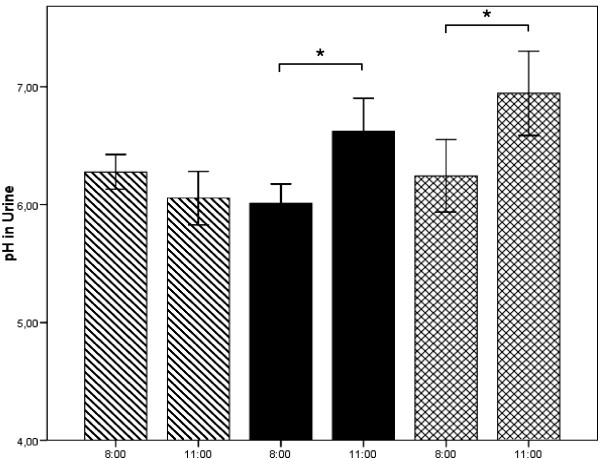
**Alterations in urinary ph without intake of the multi-mineral supplement (MMS) (hatched bars), following MMS in the morning without (black bars) or with prior 1 week of chronic MMS supplementation (crossed bars)**. Values are mean values ± SEM. * = p < .05; ** = p < .01 compared to baseline value.

The changes in urinary pH in the morning are depicted in Fig. 3. Significant alterations were not observed without MMS (hatched bars). Following MMS, urinary pH was significantly higher in the 3 hour collection period than in the collection period before supplementation (black bars and crossed bars). Comparable to blood pH, urinary baseline pH at 8 a.m. was significantly higher after the 1 week supplementation period. Table 2 shows the changes in carbondioxide (pCO_2_), bicarbonate and base excess in blood. No alterations were found without MMS (Study Visit I). Following MMS ingestion in the morning (Study Visit II) pCO_2_, bicarbonate and base excess increased significantly. Following MMS ingestion after the 1 week supplementation period, the trend was comparable but only pCO_2 _at 10:00 and 11:00 a.m. were significant.

**Table 2:** Carbondioxide, standardized bicarbonate and base excess in blood.

Study Visit	Time	pCO_2 _(mmHg)	Bicarbonate (mmol/l)	Base Excess (mmol/l)
I	8:00	38,37 ± 0,81	23,63 ± 0,29	-0,95 ± 0,29
	9:00	38,48 ± 0.77	23,77 ± 0,29	-0,93 ± 0,28
	10:00	38,36 ± 0,78	23,69 ± 0,28	-0,96 ± 0,28
	11:00	38,52 ± 0,81	23,81 ± 0,31	-0,92 ± 0,27
				
II	8:00	38,36 ± 0,8	23,58 ± 0,31	-0,99 ± 0,23
	9:00	39,75 ± 0,75*	24,74 ± 0,29**	0,14 ± 0,22**
	10:00	39,45 ± 0,68*	24,58 ± 0,26**	0,1 ± 0,24**
	11:00	39,73 ± 0,66*	24,52 ± 0,26**	-0,2 ± 0,23**
				
III	8:00	36,96 ± 0,63	23,21 ± 0,29	-1,02 ± 0,28
	9:00	38,34 ± 0,65	24,39 ± 0,28	0,11 ± 0,28
	10:00	39,84 ± 0,77*	24,62 ± 0,30	0,28 ± 0,26
	11:00	39,22 ± 0,61**	24,53 ± 0,31	0,14 ± 0,27

Study Visit I: Without MMS; Study Visit II: MMS at 8:00 in the morning without prior 1 week supplement intake twice daily; Study Visit III: MMS at 8:00 with prior 1 week chronic supplement intake twice daily. Values are mean values ± SEM. * = p < .05; ** = p < .01 compared to baseline value.

**Figure 4:**
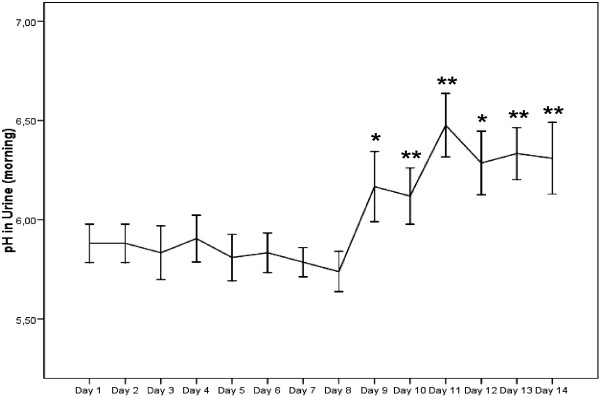
**Self-monitored urinary pH in the morning without (day 1–day 7) and with MMS (day 8–day 14)**. Values are mean values ± SEM. * = p < .05; ** = p < .01 compared to baseline value (day 1).

**Figure 5:**
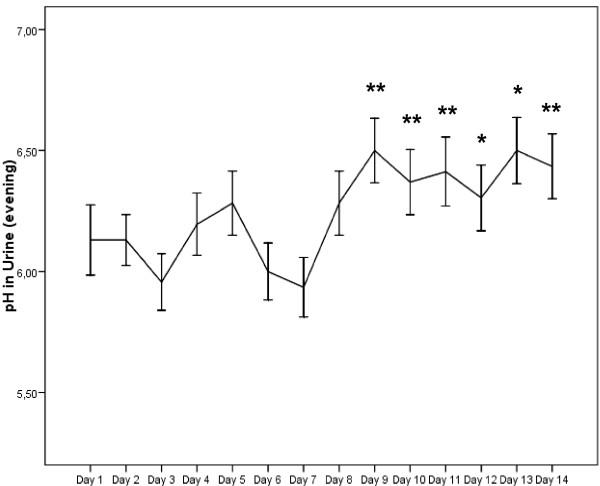
**Self-monitored urinary pH in the evening without (day 1–day 7) and with MMS (day 8–day 14)**. Values are mean values ± SEM. * = p < .05; ** = p < .01 compared to baseline value (day 1).

The results regarding self-monitored urinary pH in the morning and evening following the longer-term MMS are shown in Fig. 4 and 5. In the week without MMS, average urinary pH was 5.83 ± 0.08 in the morning and 6.11 ± 0.11 in the evening. No significant alterations from baseline value were observed. Within 24 hours, urinary pH increased significantly and remained elevated for the rest of the examination period. During the MMS mean pH in the morning was 6.28 ± 0.12 and 6.42 ± 0.13 in the evening. Also the increase in mean pH over the examination period was significantly different from mean pH without MMS (p < 0.01).

Table 3 demonstrates that dietary intakes of macronutrients, minerals and trace elements were not significantly different between the two intervention periods. Dietary potential renal acid load (PRAL = 0.49 × protein (g/d) + 0.037 × phosphorus (mg/d) - 0.021 × potassium (mg/d) - 0.026 × magnesium (mg/d) - 0.013 × calcium (mg/d)) was slightly positive and comparable during both interventions when the alkaline minerals of the supplements were not included in the equation. Inclusion of the alkaline minerals decreased PRAL levels from 5.4 ± 12 mEq to -17.5 ± 11.9 mEq.

**Table 3:** Dietary intake

	Week without Supplement	Week with Supplement	Week with Supplement*
Fat (%)	33,3 ± 1,19	33,5 ± 1,14	33,5 ± 1,14
Carbohydrate (%)	46,8 ± 1,69	46,0 ± 1,79	46,0 ± 1,79
Protein (%)	15,7 ± 0,49	16,2 ± 0,65	16,2 ± 0,65
Water (l)	2,27 ± 0,62	2,23 ± 0,58	2,23 ± 0,58
Sodium (g)	2,83 ± 1,03	2,82 ± 0,85	2,62 ± 0,85
Potassium (g)	2,97 ± 0,75	3,59 ± 0,67	2,99 ± 0,67
Calcium (g)	0,91 ± 0,26	1,48 ± 0,27	0,98 ± 0,27
Magnesium (g)	0,35 ± 0,08	0,58 ± 0,10	0,38 ± 0,10
Phosphorus (g)	1,39 ± 0,38	1,45 ± 0,04	1,45 ± 0,04
Zinc (mg)	12,8 ± 0,51	17,9 ± 0,46	12,9 ± 0,46
Copper (mg)	2,55 ± 0,18	3,51 ± 0,19	2,51 ± 0,19

Dietary intake calculated from daily nutritional protocols during the first week without intake of the mineral supplement and during the second week when the mineral supplement was ingested. Significant differences were not found. (* = The last coloumn shows dietary intake in the week with supplement minus minerals and trace elements provided by the supplement itself)

No significant changes were observed in salivary pH measured both in the morning and in the evening (data not shown).

## Discussion

The main finding of the present study was that the intake of this supplement rich in alkaline minerals was associated with a significant increase in blood and urinary pH. The small, albeit significant alkalisation of peripheral blood was already detectable following one hour after after consumption of the supplementation. The concomitant increase in plasma bicarbonate, pCO_2 _as well as the return of the slightly negative base excess to zero suggests that the mineral supplement had in fact metabolically influenced acid base balance.

Following the longer-term supplementation period, both, the increase in urinary pH in the morning and in the evening occurred within 1 day. Compared to the non-supplemented pH values, average pH in urine was 11% higher in the morning and 5% higher in the evening during the supplementation period. The data from the food records show that the increase in urinary pH was not related to any dietary change. It was an interesting finding that salivary pH did not change throughout the one week supplementation period. Our results suggest that salivary pH measurement, in contrast to determination of urinary pH, may not be an appropriate method to observe short term subtle changes in acid-base balance.

It should be noted that none of the study participants had any chronic disease or acid base imbalance. The fact that urinary pH increased following supplementation suggests that the subjects were not alkaline depleted. Nevertheless, the dosage provided enough additional alkaline minerals to improve alkaline mineral related processes of acid buffering.

Several studies have shown that increased dietary intake of alkaline mineral supplementation reduced the risk for several chronic disease conditions, mainly osteoporosis [11,19,20]. The aim of this study was not to prove the hypothesis if the intake of this supplement was associated with an improvement in any of these diseases and, therefore, no specific markers were analysed in blood or urine. Thus, it is speculative, if the evidenced alkalisation of blood and urine could have an impact on any of these chronic degenerative diseases. In view of the fact that the acidogenic properties and the resulting health problems of the prevailing western diet are more and more accepted, it remains an intriguing opportunity to correct the acid forming nutrients by alkaline minerals. Of course, changes in dietary behaviour, such as adding more fruit and reducing animal protein intake, should always be the first option [21,22].

We acknowledge the fact that the study was not placebo-controlled. It was not possible to design a placebo with a comparable solubility and flavour. However, we believe that the examination of the same measures with and without supplementation and including acute and chronic supplementation with dietary intake staying stable may, along with the unequivocal results, partly compensate for this weakness.

In conclusion, the results this study have shown that a supplementation with alkaline minerals was associated with a significant and rapid increase in blood and urinary pH and a long term increase in urinary pH after 1 week of supplementation. The health related issues of these findings remain to be determined in future studies.

## Competing interests

The authors declare that they have no competing interests.

## Authors' contributions

DK, AB, and PD conceived the idea for this study and participated in the design and coordination. DK and KM carried out the experiments and coordinated the collection of data. DK and PD performed the statistical analyses. PD, DK and AB helped for synthesis of the results and to draft the manuscript. HHD participated in study design and coordination. All authors read and approved the final manuscript.
